# Familial determinants of current smoking among adolescents of Lithuania: a cross-sectional survey 2014

**DOI:** 10.1186/s12889-015-2230-3

**Published:** 2015-09-14

**Authors:** Apolinaras Zaborskis, Dainora Sirvyte

**Affiliations:** Lithuanian University of Health Sciences, Academy of Medicine, Faculty of Public Health, A.Mickeviciaus street 9, Kaunas, LT-44307 Lithuania; Public Health Bureau of Ukmerge municipality, Deltuvos street 17, Ukmerge, LT-20127 Lithuania

## Abstract

**Background:**

Understanding the role of the family in shaping adolescent health risk behaviours has recently been given increased attention. This study investigated association between current smoking and a range of familial factors in a representative sample of Lithuanian adolescents.

**Methods:**

Study subjects (N = 3696) were adolescents aged 13- and 15-years from the schools in Lithuania who were surveyed in Spring 2014 according to the methodology of the cross-national Health Behaviour in School-aged Children (HBSC). A standard HBSC international questionnaire was translated into Lithuanian and used anonymously to obtain information about current smoking patterns and family life (family structure, quality of communication in family, parental monitoring, bonding, parenting style, family time, etc.). Logistic regression was used to assess association between smoking and familial variables.

**Results:**

The prevalence of current smoking was 16.5 % (20.8 % in boys and 11.9 % in girls; *P* < 0.001). Adjusting for gender, age and family affluence, adolescents from non-intact families were significantly more likely to be current smokers (OR = 2.10; 95 % CI: 1.74-2.54) compared with intact families. Five independent familial factors were significantly related to increased risk for adolescent smoking: low maternal monitoring (OR = 2.79; 95 % CI: 1.98-3.92), low satisfaction with family relationships (OR = 1.89; 95 % CI: 1.27-2.83), low school-related parental support (OR = 1.40; 95 % CI: 1.01-1.95), easy communication with the father (OR = 0.56; 95 % CI: 0.38-0.80) and often use of electronic media for communication with parents (OR = 0.66; 95 % CI: 0.50-0.88). The last two determinants showed an inverse effect than it was hypothesized.

**Conclusion:**

Higher prevalence of smoking among adolescents of Lithuania is associated with a non- intact family structure as well as weaker parental support and bonding. Family life practices are critical components to be incorporated in prevention and intervention programs for adolescent smoking in Lithuania.

## Background

Many countries around the globe are experiencing an increase in the prevalence of current smoking among youth and young adults and are now having to deal with the preventing tobacco use in this population [1]. Studies of school-aged children's lifestyle in Lithuania over the period 1994-2010 have demonstrated also a significant increase in the prevalence of smoking both among boys (11.3 % to 21.5 %) and girls (3.3 % to 14.8 %) [2]. Despite much efforts spent on health policy interventions implemented in the country as part of active tobacco control actions such as ban on tobacco advertising and ban of smoking in public places, increase of tobacco taxes, warnings on tobacco products, etc., and introduction of youth smoking prevention programs in schools, limited effects have been observed [2–4]. Experience and conclusions of research show that family-based programmes for preventing smoking in children and adolescents may attract the attention of public health specialists and public health politics and can be not only declared, but also really implemented as alternative to the first approach [5, 6].

The family provides the primary developmental and socialisation framework for children to learn and establish values and norms [7]. From the human development ecological perspective, the dimensions of family structure and interaction belong to the first level of analysis. Research concerned with the experience of the adolescent in the family has identified that both family structure and certain processes in family, especially communication with parents, have a clear influence on adolescents development, life chances and health behaviours [8–11].

The political and societal transition in Central and Eastern Europe, including Lithuania, started at the beginning of the 1990s brought the painful transformation in family life: tragically declining birth rates, an increased number of divorces, changes in household composition or family structure, etc. [12, 13]. For illustration of these changes, the official census data indicates a drastic increase in the number of extramarital births: from 7.0 in 1990 to 22.6 in 2000, and to 25.7 in 2010 per 100 births [14]. The proportions of children growing up in a nuclear family composed of a biological father and mother – intact family – has reduced over the past decade. These transformations may have affected child rearing and socialization of children. Such family is less able to control self destructive behaviour of the children, such as smoking, alcohol and drugs intake [12].

It is consequently crucial to understand how, and under what conditions, these changes in family structure and functioning may influence the development of the young person, especially in relation to health risk behaviours. The focus of this study lies in identifying the role that several familial factors play in adolescent smoking. Furthermore, protective factors associated with the interpersonal relationships between family members are to be identified. Within this area, there are analyses of the specific variables that shape the interpersonal relationships (family dynamics) built within the family setting including: communication and attachment to parents, monitoring, and disciplinary parenting styles [7]. A review of the literature identifies that there is better adjustment in children and adolescents (e.g. less risk of smoking) who reported having an open communication with their parents, or who perceived them as physically and emotionally accessible, or who felt vigilant parental monitoring [15–19]. Because research in this field among the Lithuanian adolescent population is still scarce [20–22], there is a need to investigate how much the above mentioned findings are appropriate within the Lithuanian family undergoing social transformations.

The *Health Behaviour in School-aged Children* (*HBSC*), a World Health Organization cross-national study, considers the family as one of the significant domains of adolescent life [7, 10, 11, 23, 24]. The study, which started in Lithuania in 1994, provides a realistic opportunity to explore adolescent health behaviour, including smoking habits, in the family context. The recent survey that was carried out in Spring 2014 in Lithuania included full set of optional packages developed by the *Family Culture* working group [7]. Comparing with survey in 2010, these packages include several new items (e.g. use of electronic media for communication with parents), as well as national items (e.g. every day seeing of parents).

The aim of this study was to investigate the association between current smoking and a range of familial factors in a representative sample of Lithuanian adolescents who were surveyed in the recent HBSC wave. We hypothesized that changes in family structure, weaker child-parent relationships and contact, lack of parental control, etc. would be related to higher risk of adolescent smoking.

## Methods

### Subjects and study procedures

The data analysed here were collected in the school-based, cross-sectional, anonymous survey conducted in 2014 (April – May) in Lithuania according to the methodology of World Health Organization collaborative cross-national HBSC study (more detailed information about the study is provided elsewhere [23–25]). Researchers followed the standardized international research protocol to ensure consistency in survey instruments, data collection and processing procedures.

The population selected for sampling was 11-, 13- and 15-year-olds attending general school. Participants were selected using a clustered hierarchical sampling design, where the initial sampling unit was a class of the fifth, seventh or ninth grades (the most appropriate grades for required students’ age groups). Samples of students were drawn to be representative by age and gender. The recommended sample size for each survey was approximately 1,500 students per age group. In total, 356 classes from 129 schools from the whole country were drawn to ensure the requested number of surveyed students.

Questionnaires were administered in school classrooms by form tutors who complied with written instructions. The time frame for filling out the questionnaires was 1 – 1½ school period. Participants could freely choose to participate, and anonymity and confidentially was ensured. As finishing questioning, students sealed themselves the provided envelopes with questionnaires inside. Form tutors reported about the number of participants and process of questioning. The response rate was 84 %.

Upon the completion of the fieldwork, the data were prepared using standard documentation and submitted to the HBSC International Data Bank at the Bergen University, Norway. The data were checked, cleaned, included into the international HBSC database, as well as returned to the country for further statistical processing (*N* = 5730).

The present study includes 3696 students aged 13- and 15-years and who reported about current smoking (the proportion of non-reported current smoking cases was 0.5 %, *N* = 19). The youngest group of 11-year-old adolescents (*N* = 2015) was excluded from the analysis because the proportion of current smokers in this group was relatively low (2.3 %).

### Instrument and measures

We used the standard HBSC international questionnaire adopted after its translation from the Standard English version [26] into Lithuanian and retranslated back into English. The questionnaire consists of a mandatory (obligatory) section, that each country is required to include for the production of an international HBSC database, and optional packages, e.g. an optional package "*Family Culture*" [7].

In the present study, the outcome measure was a current smoking assessment from the mandatory section. Adolescents were asked how often they smoke tobacco at present. The answer alternatives ranged: 'every day', 'at least once a week, but not every day', 'less than once a week', and 'I do not smoke'. The findings presented here are two proportions of respondents: those who reported 'I do not smoke' (not smokers), and those who reported all stages of smoking (smokers).

The list of independent variables included gender and age group (13-year-olds and 15-year-olds) of the respondent, as well as a series of the following familial variables from the mandatory section.

#### Family affluence scale

The HBSC Family Affluence Scale (FAS) measure is based on a set of questions on the material conditions of the households in which young people live [26, 27]. The questions are easy for children to answer and cover car ownership, bedroom and bathroom occupancy, holidays, home computers and dishwashing machine. A composite FAS score was calculated for each respondent based on his responses to these six items and than a three-point (low, medium and high) ordinal scale was composed for the analysis.

#### Family structure

Family was defined as 'the persons you live together with'. On the list of adult people ('father', 'step-father', 'mother', 'step-mother', and etc.), the respondents were asked to indicate the persons living in their family. The respondents who indicated both 'father' and 'mother' were included into a group of adolescents living in intact families (living with both biological parents), while all remaining respondents were considered as adolescents living in not intact families, which included lone parent families, stepfamilies or reconstituted families, and looked after children, i.e. in a foster home or children's home. The family structure measure was validated using reports to other questions that included the answer option 'I don't have or see this person'.

#### Communication with parents

In the mandatory section there were two questions about ease of communication with the respondent’s father and mother. We asked the respondents “How easy is it for you to talk to the following persons about things that really bother you?” A checklist of close people including ‘father’ and ‘mother’ was then given, with the response options of ‘very easy’, ‘easy’, ‘difficult’, ‘very difficult’ and ‘don’t have or don’t see this person’. We collapsed the original answer options of ‘very easy’ and ‘easy’ into one category, 'easy communication with …'. This was also done with the original answering options ‘very difficult’ and ‘difficult’ into 'difficult communication with…'. The decision to dichotomize the answering categories was in accordance with suggestions from the HBSC *Family Culture* focus group concerning the application of this variable in the international analyses [24]. The option 'don’t have/see this person' was used for either ‘mother’ or ‘father’, and this data indicated on respondent's living in not intact family.

#### Quality of family communication

In 2013/2014 the HBSC *Family Culture* focus group added new items - a shortened version of the clear communication scale from *Family Dynamics Measure II* [28, 29]. Four items of this scale were designed to explore quality of communication in the family. Our data showed that this scale have very good reliability (*Cronbach*'s Alpha was 0.87 both in the total sample and the subsample of intact families). In the further analysis (see *Statistical analysis*), the individual responses to items of the scale, as well as to items of other scales used in this study, were transformed into one quantitative value (factor score) and then dichotomized into positive and negative factor score values. Positive factor score values showed 'good' quality of communication in families (respondents agreed that in their families 'important things talked about', 'someone listens', 'ask questions', 'clarify misunderstanding'). In contrast, negative factor values showed 'poor' quality of communication in family.

#### Satisfaction with family relationships

This variable was measured by means of an item based on *Cantril*'s ladder [30] asking respondents people to rate their satisfaction with relationships or global atmosphere in their family. A quantitative score was obtained that ranged from 0 'we have very bad relationships in our family' to 10 'we have very good relationships in our family'. On the basis of frequency analysis of this score, the answers were dichotomized with those who considered relationships 'high' (7-10 scores) as one group and the rest of the answers as another ('low') group.

The rest familial variables were taken from the optional "*Family Culture*" package.

#### Parental monitoring

The measure of parental monitoring was based on the scale developed by *Brown* et al. [31], which asks young people about how much their father and mother (repeated for each of them) knows about five issues: "Who your friends are"; "How you spend your money"; "Where you are after school"; "Where you go at night"; and "What you do with your free time". The answer score ranged from 1 'don't know anything' to 3 'know a lot', where higher scores represents higher levels of parental knowledge about child's matters. In the scale reliability analysis *Cronbach*'s Alphas were 0.90 and 0.79 correspondingly for the father's and mother's answers. In further analyses, the undertaken 1-factor scores calculation (see below) defined two binary variables (for father and mother) with positive and negative factor score values that corresponded to the 'high' and 'low' level of parental monitoring.

#### Emotional support

This measure is 4-items subscale of the already classic instrument build by *Parker* et al. [32], which is used to assess the quality of parental bonding. Items are repeated for the father and the mother. The respondents were asked how often their father and mother "Helps me as much as I need"; "Is loving"; "Understands my problems and worries"; and "Makes me feel better when I am upset". Answering options were scored: 1 'never', 2 'sometimes', 3 'almost always'. In the scale reliability analysis *Cronbach*'s Alphas were 0.84 and 0.78 correspondingly for the father's and mother's answers. The 'high' and 'low' emotional support were defined as corresponding positive and negative factor score values derived from 1-factor analysis of the subscale items.

#### Promotion of autonomy

Four items of this measure consist the second dimension of *Parker*'s et al. [32] parental bonding inventory. The respondents were asked how often their father and mother "Lets me do the things I like doing"; "Likes me to make my own decisions"; "Tries to control everything I do"; and " Treats me like a baby". Answering options and further processing of this measure were the same as for emotional support measure. In addition, the responses to the 3-rd and 4-th items were reversely coded to create a gradient with higher scores indicating a more positive attitude towards promotion of autonomy. Because of low reliability (*Cronbach*'s Alphas were 0.55 and 0.40 correspondingly for the father's and mother's answers) this subscale of parental bonding was not used in the further analysis.

#### Family time together

The evaluation of joint family activity was based on 8 items: (1) watching TV or a video, (2) playing indoor games, (3) eating meals, (4) going for a walk, (5) going places, (6) visiting friends or relatives, (7) playing sports, (8) sitting and talking about things. Respondents were asked how often did they do any of these activities and spend time together in shared activities. The response options ranged from 'never' (coded as 1) to 'every day' (coded as 5). In 1-factor analysis of the scale using the method described below a binary variable was created. Two categories of this variable 'often' and 'rare' corresponded to more often and less often respondent's suggestion of doing several joint family activities. Reliability of the family time together scale was assessed as very good (*Cronbach*'s Alpha was 0.85 both in the total sample and the subsample of intact families).

#### School-related parental support

The rationale of this measure is based on the concepts of school setting developed previously in the HBSC surveys [33]. Although each item of the scale has a concern in the parental support in various aspects of school the scale measures broad perceptions of parental emotional support and parental controlling. The students were asked to show how they agree or disagree with the following 5 statements: (1) "If I have a problem at school, my parents are ready to help"; (2) "My parents are willing to come to school to talk to teachers"; (3) "My parents encourage me to do well at school"; (4) "My parents are interested in what happens to me at school"; and (5) "My parents are willing to help me with my homework". The scores from 1 'strongly disagree' to 5 'strongly agree' were obtained from this scale and processed in 1-factor analysis. A binary variable was created, which category 'high' indicates on student's willingness to agree with positive parental support for his school-related issues, while the category 'low' indicates the reverse. Our data showed very good reliability of the school-related parental support scale (*Cronbach*'s Alpha was 0.85 both in the total sample and the subsample of intact families).

#### Parenting style

This measure refers to the strategies that parents used for the socialization of their children. HBSC includes 4 items in optional section to measure this topic. According to *Maccoby* and *Martin* [34], the scale measures at once the four well-known parental disciplinary styles: (1) authoritative-reciprocal; (2) permissive-indulgent; (3) authoritarian-repressive; and (4) permissive-neglectful. The previous surveys demonstrated a high reliability of the scale and, however, a big overlap between styles. In the present HBSC survey, to clarify disciplinary styles we changed the scale to one question that was repeated for the father and the mother. Young people were asked: "What does your father/mother does, when you do something that he/she thinks is wrong?" The respondents could chose one of the 4 response options: (1) "My father/mother explains to me what I have done wrong and why I am being punished"; (2) "My father/mother tells me that I behaved badly but doesn't punish me"; (3) "My father/mother punishes me immediately without telling me why"; and (4) "My father/mother doesn't punish me, he/she takes no notice". Each of these alternatives indicates to corresponding parental disciplinary style listed above.

#### Electronic media communication with parents

A new measure for the 2013/2014 HBSC survey was developed to assess how often children communicate with parents using phone and computers. In the national questionnaire, adolescents were asked to answer how often, in average, they communicate with parents in these ways: (1) speaking by phone; (2) sending SMS messages; (3) writing e-letters; and (4) conversing in real time (e.g. by *Skype*). Response options were scored: 1 'never'; 2 'once per week'; 3 'several times per week'; 4 'once a day'; and 5 'several times a day'. The *Cronbach*'s Alphas of the scale was 0.61. The 'often' and 'rare' electronic media communication with parents were defined as corresponding positive and negative factor score values derived from 1-factor analysis of the subscale items.

#### Seeing of parents

A national item on how often children are able to see (meet) their parents because of their job was developed. The item was repeated for the father and the mother. In analyses, the 5 response options of the question were collapsed into two groups: (1) seeing father/mother every day, and (2) seeing father/mother not every day (combined options: 'few days per week'; 'couldn't see for several weeks'; 'haven't seen more than a month'; and 'haven't seen more than a year').

### Statistical analysis

Data were analysed in two steps. The first step of analysis was performed within the total sample of 13- and 15-year-olds (*N* = 3696), in order to assess the relationship between current smoking and family structure only. The second step of analysis was performed within the subsample of those living in intact families (*N* = 2528), and was aimed to explore relationships between current smoking and a set of variables specific for the intact family.

Reliability analysis with *Cronbach*'s Alpha measure was used to establish the level of internal consistency of various multi-item scales. Explanatory 1-factor analysis with a principal component analysis was adopted for each scale to construct reliable one-dimensional variables. The factor scores were calculated within subsample of intact families in such way that higher factor scores indicated a higher/better level of family life expected by the respondents. Next, using 0 as a cut-point, factor score values were dichotomized into positive and negative groups, which corresponded to respondents' inclination for higher and lower scores in the scale.

Associations between familial measures and current smoking were estimated using odds ratios (OR) with 95 % confidence intervals (95 % CI) in a binary logistic regression analysis. We used Enter method in multivariable analyses with all variables irrespective of their significance found in a univariable analysis. Interactions between familial variables were tested. All analyses were performed with SPSS (version 20.0; SPSS Inc, Chicago, IL, 2010). *P* < 0.05 was considered statistically significant.

### Ethics

The study was conformed to the principles outlined in the Declaration of Helsinki. Ethical approval for the study was granted by the Kaunas Regional Biomedical Research Ethics Committee (reference number BE-2-16). In line with local practice for general school surveys, the study was agreed with national and local educational institutions. Additionally, written informed consent for participation in the study was sought from parents.

## Results

The study sample was balanced by gender and age groups, while about two-thirds (62.8 %) of the total sample of respondents were regarded as living in intact families. Table 1 shows demographic and parental characteristics of all studied adolescents and subsample of adolescents living in intact families. For the items repeated for the father and the mother, there was a significant difference in respondents' opinion about father's and mother's role in their life. Easy communication with the mother was reported more often than with the father (75.9 % vs 63.1 %; *P* < 0.001). High level of maternal monitoring was more prevalent than paternal monitoring (61.8 % vs 49.3 %; *P* < 0.001). According to the adolescents' opinion, they can get high emotional support from their mothers more often than from fathers (61.5 % vs 57.1 %; *P* = 0.002). Authoritative-reciprocal parenting style the mothers showed more often than the fathers (46.2 % vs 42.2 %; *P* < 0.001). Using telecommunication technology, children preferred to converse with their mothers more often than with fathers (75.9 % vs 63.1 %; *P* < 0.001).

**Table 1:** Demographic and parental characteristics of the studied total sample and subsample

Predictors	Number	Percent	*P* ^a^
Total sample, *N* = 3696			
Gender:			
Boys	1888	51.1	
Girls	1808	48.9	
Age:			
13-year-old	2004	54.2	
15-year-old	1692	45.8	
Family FAS:			
Low	1322	36.9	
Medium	1567	43.7	
High	696	19.4	
Family structure:			
Intact family	2528	68.6	
Not-intact family	1158	31.4	
Subsample of respondents living in intact families, *N* = 2528			
Gender:			
Boys	1290	51.0	
Girls	1238	49.0	
Age:			
13 years old	1392	55.1	
15 years old	1136	44.9	
Family FAS:			
Low	779	31.7	
Medium	1110	45.2	
High	566	23.1	
Satisfaction with family relationships:			
High	2144	86.2	
Low	342	13.8	
Communication with the father:			
Easy	1484	63.0	<0.001
Difficult	870	37.0	
Communication with the mother:			
Easy	1791	75.8	
Difficult	572	24.2	
Quality of communication in the family:			
Good	1567	62.5	
Poor	941	37.5	
Father's monitoring:			
High	1234	49.2	<0.001
Low	1274	50.8	
Mother's monitoring:			
High	1549	61.8	
Low	959	38.2	
School-related parental support:			
High	1294	51.6	
Low	1214	48.4	
Family time together:			
Often	1177	46.7	
Rare	1345	53.3	
Father's emotional support:			
High	1445	57.2	0.002
Low	1083	42.8	
Mother's emotional support:			
High	1555	61.5	
Low	973	38.5	
Father's parenting style:			
Authoritative- Reciprocal	1045	42.2	<0.001
Permissive-indulgent	1017	41.2	
Authoritarian-repressive	194	7.8	
Permissive-neglectful	218	8.8	
Mather's parenting style:			
Authoritative-reciprocal	1147	46.1	
Permissive-indulgent	1085	43.6	
Authoritarian-repressive	158	6.4	
Permissive-neglectful	98	3.9	
Electronic media communication with parents			
Often	1099	43.5	
Rare	1428	56.5	
Seeing of the father			
Every day	1912	77.4	<0.001
Not every day	559	22.6	
Seeing of the mother			
Every day	2333	94.0	
Not every day	148	6.0	

^a^Significant difference in respondents' opinion about the father and the mother (Chi-squared test)

In the total sample of 13- and 15-year-old adolescents, the prevalence of current smoking (daily, weekly, less than weekly) was 16.5 %, higher in boys than in girls (20.8 % vs 11.9 %; *P* < 0.001), and increasing from 10.7 % to 23.3 % (*P* < 0.001) at two years of age. Smoking was found to be significantly associated with family structure: with lower smoking prevalence among adolescents in intact families and higher smoking prevalence among adolescents in not-intact families (crude OR = 2.13, 95 % CI: 1.78-2.54; and adjusted by gender, age and family FAS, OR = 2.10, 95 % CI: 1.74-2.54) (Table 2). Comparing the groups of boys and girls, there was no significant difference in this association. Adolescents from medium and high family FAS groups had lower odds to be current smokers comparing with adolescents from low family FAS group.

**Table 2:** Current smoking among 13 and 15 year old adolescents by gender, age, and family structure (total sample, *N* = 3696)

Predictors	Non-smokers *n* (%)	Smokers *n* (%)	Univariate logistic regression		Multivariate logistic regression	
			*OR*	*CI*	*OR*	*CI*
Gender:						
Boys	1495 (79.2)	393 (20.8)	1.00	Ref.	1.00	Ref.
Girls	1592 (88.1)	216 (11.9)	**0.52**	**0.43–0.62**	**0.53**	**0.44–0.64**
Age:						
13-year-old	1789 (89.3)	215 (10.7)	1.00	Ref.	1.00	Ref.
15-year-old	1298 (76.7)	394 (23.3)	**2.53**	**2.11–3.03**	**2.55**	**2.11–3.08**
Family FAS:						
Low	1069 (80.9)	253 (19.1)	1.00	Ref.	1.00	Ref.
Medium	1336 (85.3)	231 (14.7)	**0.73**	**0.60–0.89**	**0.80**	**0.65–0.98**
High	599 (86.1)	97 (13.9)	**0.68**	**0.53–0.88**	0.86	0.67–1.12
Family structure:						
Intact family	2201 (87.1)	327 (12.9)	1.00	Ref.	1.00	Ref.
Not-intact family	880 (76.0)	278 (24.0)	**2.13**	**1.78-2.54**	**2.10**	**1.74–2.54**

Significant relationships are provided in bold

Univariable logistic regression analysis (Table 3) showed that smoking of adolescents living in intact families was significantly associated with the following familial factors: low satisfaction with family relationships; difficult communication with the mother; low parental monitoring; poor quality of communication in the family; low school-related parental support; rare family time together; low parental emotional support; authoritarian-repressive parenting style of both parents and permissive-neglectful parenting style of the father; not every day seeing of the mother.

**Table 3:** Possible familial predictors of current smoking among 13 and 15 year old adolescents (subsample of respondents living in intact families, *N* = 2528)

Predictors	Non-smokers *n* (%)	Smokers *n* (%)	Univariable logistic regression		Multivariable logistic regression^a^	
			*OR*	*CI*	*OR*	*CI*
Gender:						
Boys	1075 (83.3)	215 (16.7)	1.00	Ref.	1.00	Ref.
Girls	1126 (91.0)	112 (9.0)	**0.50**	**0.39–0.63**	**0.61**	**0.45–0.83**
Age:						
13-year-old	1283 (92.2)	109 (7.8)	1.00	Ref.	1.00	Ref.
15-year-old	918 (80.8)	218 (19.2)	**2.80**	**2.19–3.57**	**2.58**	**1.92–3.45**
Family FAS:						
Low	665 (85.4)	114 (14.6)	1.00	Ref.	1.00	Ref.
Medium	985 (88.7)	125 (11.3)	**0.74**	**0.56–0.97**	0.81	0.56–1.18
High	493 (87.1)	73 (12.9)	0.86	0.63-1.19	1.15	0.83-1.60
Satisfaction with family relationships:						
High	1899 (88.6)	245 (11.4)	1.00	Ref.	1.00	Ref.
Low	268 (78.4)	74 (21.4)	**2.14**	**1.60–2.86**	**1.89**	**1.27–2.83**
Communication with the father:						
Easy	1281 (86.3)	203 (13.7)	1.00	Ref.	1.00	Ref.
Difficult	774 (89.0)	96 (11.0)	0.78	0.60–1.02	**0.56**	**0.38–0.80**
Communication with the mother:						
Easy	1577 (88.1)	214 (11.9)	1.00	Ref.	1.00	Ref.
Difficult	485 (84.8)	87 (15.2)	**1.32**	**1.01–1.73**	1.18	0.81–1.71
Father's monitoring:						
High	1123 (91.0)	111 (9.0)	1.00	Ref.	1.00	Ref.
Low	1059 (83.1)	215 (16.9)	**2.05**	**1.61–2.62**	1.19	0.83–1.69
Mother's monitoring:						
High	1430 (92.3)	119 (7.7)	1.00	Ref.	1.00	Ref.
Low	752 (78.4)	207 (21.6)	**3.31**	**2.60–4.22**	**2.79**	**1.98–3.92**
Quality of communication in the family:						
Good	1395 (89.0)	172 (11.0)	1.00	Ref.	1.00	Ref.
Poor	787 (83.6)	154 (16.4)	**1.59**	**1.26–2.01**	0.99	0.71–1.38
School-related parental support:						
High	1180 (91.2)	114 (8.8)	1.00	Ref.	1.00	Ref.
Low	1002 (82.5)	212 (17.5)	**2.19**	**1.61–2.92**	**1.40**	**1.01–1.95**
Family time together:						
Often	1046 (89.6)	122 (10.4)	1.00	Ref.	1.00	Ref.
Rare	1136 (84.3)	204 (15.2)	**1.54**	**1.21–1.96**	0.98	0.71–1.35
Father's emotional support:						
High	1296 (89.7)	149 (10.3)	1.00	Ref.	1.00	Ref.
Low	905 (83.6)	178 (16.4)	**1.71**	**1.35–2.16**	1.36	0.96–1.91
Mother's emotional support:						
High	1384 (89.0)	171 (11.0)	1.00	Ref.	1.00	Ref.
Low	817 (84.0)	156 (16.0)	**1.55**	**1.22–1.95**	0.72	0.51–1.01
Father's parenting style:						
Authoritative-reciprocal	931 (89.1)	114 (10.9)	1.00	Ref.	1.00	Ref.
Permissive-indulgent	896 (88.1)	121 (11.9)	1.10	0.84–1.45	0.78	0.45–1.33
Authoritarian-repressive	157 (80.9)	37 (19.1)	**1.93**	**1.28–2.89**	0.80	0.47–1.35
Permissive-neglectful	177 (81.2)	41 (18.8)	**1.89**	**1.23–2.80**	1.21	0.64–2.28
Mother's parenting style:						
Authoritative-reciprocal	1018 (88.8)	129 (11.2)	1.00	Ref.	1.00	Ref.
Permissive-indulgent	939 (86.5)	146 (13.5)	1.23	0.95–1.59	1.07	0.49–2.35
Authoritarian-repressive	130 (82.3)	28 (17.7)	**1.70**	**1.09–2.66**	1.20	0.55–2.68
Permissive-neglectful	82 (83.7)	16 (16.3)	1.54	0.87–2.71	0.91	0.37–2.24
Electronic media communication with parents:						
Often	943 (85.8	156 (14.2)	1.00	Ref.	1.00	Ref.
Rare	1258 (88.0)	171 (12.0)	0.82	0.66-1.04	**0.66**	**0.50-0.88**
Seeing of the father:						
Every day	1667 (87.2)	245 (12.8)	1.00	Ref.	1.00	Ref.
Not every day	492 (88.0)	67 (12.0)	0.93	0.70–1.24	1.20	0.85–1.70
Seeing of the mother:						
Every day	2045 (87.7)	288 (12.3)	1.00	Ref.	1.00	Ref.
Not every day	120 (81.1)	28 (18.9)	**1.66**	**1.08–2.55**	0.90	0.53–1.53

^a^Method = Enter. Significant relationships are provided in bold

In a multivariable logistic regression analysis (Table 3), adjusting data for gender, age and family FAS, five independent familial factors were significantly related to increased risk for adolescent smoking: low mother's monitoring (OR = 2.79; 95 % CI: 1.98-3.92), low satisfaction with family relationships (OR = 1.89; 95 % CI: 1.27-2.83), low school-related parental support (OR = 1.40; 95 % CI: 1.01-1.95), while easy communication with the father (OR = 0.56; 95 % CI: 0.38-0.80) and often telecommunication with parents by phone or computer (OR = 0.66; 95 % CI: 0.50-0.88) were not found as protective factors.

Results from multivariable logistic regression in Table 4 shows differences between boys and girls in assessing importance of familial factors to risk for adolescent current smoking. Both for the boys and girls, high maternal monitoring was identified as a protective factor but stronger among girls, and easy communication with the father showed an inverse effect. The inverse effect of often electronic media communication with parents was observed among the boys only. For the girls, high satisfaction with family relationships, high school-related parental support, and high father's emotional support were identified as significant smoking protective factors too.

**Table 4:** Familial predictors of current smoking among 13 and 15 year old boys and girls (subsample of respondents living in intact families, results from multivariable logistic regression^a^)

Predictors^b^	Boys (*N* = 1290)		Girls (*N* = 1238)	
	*OR*	*CI*	*OR*	*CI*
Age:				
13-year-old	1.00	Ref.	1.00	Ref.
15-year-old	**2.56**	**1.76–3.72**	**2.87**	**1.75–4.70**
Family FAS:				
Low			1.00	Ref.
Medium			**0.41**	**0.23–0.77**
High			0.73	0.41–1.28
Satisfaction with family relationships:				
Good			1.00	Ref.
Poor			**2.20**	**1.19–4.05**
Communication with the father:				
Easy	1.00	Ref.	1.00	Ref.
Difficult	**0.59**	**0.35–0.98**	**0.47**	**0.27–0.83**
Mother's monitoring:				
High	1.00	Ref.	1.00	Ref.
Low	**2.39**	**1.55–3.70**	**4.16**	**2.37–7.29**
School-related parental support:				
High			1.00	Ref.
Low			**2.49**	**1.34–4.63**
Father's emotional support:				
High			1.00	Ref.
Low			**1.83**	**1.01–3.35**
Electronic media communication with parents:				
Often	1.00	Ref.		
Rare	**0.69**	**0.48–0.95**		

^a^Method = Enter; ^b^Results of all significant predictors are provided. Significant relationships are provided in bold

## Discussion

This paper draws on recent Lithuanian data from the World Health Organization cross-national HBSC study, which investigates a range of familial determinants on youth health and heath behaviour [7]. We aimed to investigate the association between current smoking and several familial factors among adolescents. Smoking prevalence was the main focus due to increasing prevalence of this risk-taking behaviour among Lithuanian adolescents [2, 3].

The associations between parental or familial factors and adolescent risk-taking behaviour have been extensively examined [17–19, 35–38]. Various variables to describe family functioning and parenting have received a great deal of attention [7, 39, 40]. However, in this study socio-economic factors as well as measures of parental knowledge, attitudes and behaviour were not analyzed. We assessed the statistical significance of associations with current smoking for at least 15 determinants, which measured different aspects of child-parent relationships. In order to avoid effect overestimation of the father's or mother's role in single-parent and step-parent families, an intact family was selected as a model to obtain valid findings. Partly, this decision was supported by *Recker'*s research [41].

The rationale for this study arose from the significant family transformations over last two decades that were the consequence of a swift transition away from a totalitarian regime to a democratic society in Central and Eastern European countries, including Lithuania. The family across Eastern Europe is undergoing rapid social change and warrants detailed examination [12].

The present study revealed that only 62.8 % of the total sample of respondents were living in intact families, whereas two decades ago, in 1994, during the first HBSC study wave in Lithuania, the corresponding figure was significantly greater – 82.7 % [42]. Family structure has been previously linked to the risk of engaging adolescents in smoking [18, 43]. The results of our study were in accordance with the literature indicating that adolescents who did not live with both parents were more than twice more likely to smoke than their peers who lived in an intact family. Although in Lithuania smoking is more common among boys [2, 24, 42], the association was equally strong both among boys and girls. In 1994, the association between smoking and family composition was significant too but much weaker than was found for 2014 [42]. The presented change in the value of association indicates to some processes manifesting in a family as a consequence of transition period and acting more expansive than destruction of the family composition. We hypothesize that such processes could initiate trends in family functioning and communication patterns.

It is well known that conflicts in good parent-child relations and communication is a key predictor of poor psychological well-being and behavioural problems among young people and it is found that good communication with mother and with father is associated with positive outcomes in relation to smoking and other risk-taking behaviours [15–19]. Our previous studies which were based on the data of HBSC surveys in 2002 [21] and 2006 [22] confirmed that such association is a consistent pattern for both boys and girls and with respect to both parents. In in these studies we found that easy communication with parents is a more robust barrier to smoking than living with both parents, attachment to the mother being particularly important for girls aged 15 years. In line with other studies [11] and our previous studies [21, 22, 42], the present study showed that adolescents find it easier to talk to their mother rather than to their father. We confirmed (in univariable analysis) easy talking to mother as a protective factor against adolescent smoking. But it was no longer protective factor in regard to easy talking to father. In multivariable analysis, particularly among girls, the association between easy talking to father and smoking was found inverse. However, herein we found a small positive association between father's emotional support of daughters and less risk for their smoking.

The protective role of parental monitoring against health risk behaviours among adolescents has been widely discussed [19]. Several studies have identified that both paternal and maternal monitoring is the strongest predictor of outcome adolescent risk-taking behaviours [17, 44, 45]. In line with these studies, our study also supported such findings, in addition the current study also stresses the importance of mother's role in prevention of smoking among adolescents by strengthening their monitoring in family.

It was hypothesized that frequent interactions with parents by phone or using other electronic media can facilitate positive communication with parents, as well as can play a helpful role in the monitoring of children. Consequently, frequent communication should play a protective role [7]. However, in contrast the findings of our study indicated that frequent communication with parents using electronic media increased the risk of current smoking in adolescents by about 1.5 times.

It is not easy to explain these relatively new results concerning changes in the association between parent roles and Lithuanian adolescent smoking. Some interactions between presented findings, however, could be distinguished. There could be a presumption that democratization of family life with less authoritarian parenting became more common in Lithuanian family life, particularly in father-child relationships. Consequently, this process might decrease adolescent fear of being punished by his father due to smoking or some other inappropriate behaviour, and hereafter, facilitate talks with him face-a-face or using electronic media. In these parenting constructs, it does not matter how much the father can know where and what the child is doing, e.g. how strongly the adolescent is being on paternal monitoring. Therefore, there is a need for further research to confirm these associations as no other studies investigating such associations were found.

### Study strengths and limitations

The current study has several strengths and limitations.

The high representativeness of the sample selected and high participation rate in the survey could be considered as the strengths of primary importance of the current study. It is also important that our research was a part of the cross-national collaborative HBSC study. The application of standardized methods including the HBSC questionnaire, which was developed by international experts, is another advantage of this study. The measures of family life were based on valid scales. The internal reliabilities (*Cronbach*'s alphas) for the applied scales were high. The results of this type of research is also a step forward to filling the gap of mapping health inequalities in the context familial determinants of substance abuse in youth.

Some limitations of our study are related to an inherent problem as using the self-reported data could introduce some biases. Our questionnaire survey as well as other similar studies carried on adolescents health behaviour presents an example of very sensitive and personal issues for investigation [46]. To cope with this source of a potential bias of self-reporting, special attempts were made by researchers to provide warranty of anonymity and confidentiality. In addition, the questions were subject to piloting and pre-testing at international and national levels prior to the main survey [7, 47].

From the data analysis perspective the analysis of associations between smoking and familial variables in the current study is limited within intact families, in general. The reasons for such approach were methodological constraints that limited application of selected measures in non-intact families if they were specified for the father and the mother. For instance, the data of the present survey demonstrated that 1046 (28.4 %) respondents were living in a family without the father, therefore, in this group 426 (40.7 %) respondents reported how easy is it to communicate with their fathers, or 633 (60.5 %) respondents indicated how much their fathers know about their activities. Such disparities can be naturally occurred as the family is divorced, but the child is able to meet his father, for example. However, the described cases are confusing in regard of the simple definition of family structure and the further studies are need to explore non-intact families.

The study did not include information related to parents' and peers' cigarette smoking, or second hand smoke exposure at home or other places; which may play an important role in determining current smoking of young people, as the survey did not include related information. Nevertheless, our study focussed on the specific psychosocial familial determinants and their impact on young people smoking risk, and results provide baseline information in given context, which provide directions for improved tobacco control efforts intervening at family level.

Finally, given the cross-sectional design of this study with a rather exploratory nature, we should be careful with interpreting causality. Thus, more studies, including studies with a longitudinal design, are needed to confirm the results and to establish scientific evidence on the relationships found in this study. If these results are confirmed, parents should be advised to apply the more positive approach in parenting and managing their parental roles by helping their children to achieve certain goals.

## Conclusions

Overall, the findings suggest that prevalence of smoking among adolescents of Lithuania is associated with an intact family structure as well as weaker parental support and bonding. However maternal monitoring is a particularly important protective factor, particularly among girls, while the father's role seems to be diminishing in changing society. Results show that family life practices are critical components to be incorporated in prevention and intervention programs for adolescent smoking in Lithuania.

## Abbreviations

CI: Confidence interval
FAS: Family affluence scale
FDM II: Family dynamics measure II
HBSC: Health Behaviour in School-aged Children, a World Health Organization cross-national study
OR: Odds ratio
